# Neighborhood Environment and Objectively Measured Sedentary Behavior Among Older Adults: A Cross-Sectional Study

**DOI:** 10.3389/fpubh.2020.552198

**Published:** 2021-01-12

**Authors:** Shao-Hsi Chang, Ru Rutherford, Ming-Chun Hsueh, Yi-Chien Yu, Jong-Hwan Park, Sendo Wang, Yung Liao

**Affiliations:** ^1^Department of Physical Education, National Taiwan Normal University, Taipei, Taiwan; ^2^Department of Health Promotion and Health Education, National Taiwan Normal University, Taipei, Taiwan; ^3^Graduate Institute of Sport Pedagogy, University of Taipei, Taipei, Taiwan; ^4^Health Convergence Medicine Laboratory, Biomedical Research Institute, Pusan National University Hospital, Busan, South Korea; ^5^Department of Geography, National Taiwan Normal University, Taipei, Taiwan

**Keywords:** sedentary behavior pattern, environment, accelerometer, urban older adults, walkability

## Abstract

**Background:** We examined the relationships between objectively assessed neighborhood environment and the patterns of sedentary behavior among older adults.

**Methods:** A total of 126 community-dwelling older adults (aged 65 years or above) were recruited. Data on neighborhood environmental attributes (resident density, street intersection density, sidewalk availability, accessible destinations, and accessible public transportation), accelerometer-assessed total time and patterns of sedentary behavior (number and duration of bouts), and sociodemographic characteristics were collected. Multiple linear regression models were developed.

**Results:** After adjustment for potential confounders, greater sidewalk availability was negatively related to the number of sedentary bouts (β = −0.185; 95% CI: −0.362, 0.015; *p* = 0.034) and sedentary bout duration (β = −0.180; 95% CI: −0.354, −0.011; *p* = 0.037).

**Conclusions:** This study revealed that a favorable neighborhood environment characterized by sidewalk availability is negatively associated with sedentary behavior patterns in Taiwanese older adults. These findings are critical to inform environmental policy initiatives to prevent sedentary lifestyle in older adults.

## Introduction

As is the case with many countries around the world, the population of older adults is increasing rapidly in Taiwan. In 2018, older adults accounted for 14.05% of the total population, and Taiwan will become a super-aged society by 2026 (1). Maintaining a healthy lifestyle is a key determinant of older adults' health (2, 3). It is well-documented that older adults should engage in sufficient levels of physical activity in order to obtain substantial health benefits (4). In addition to physical activity, emerging evidence has shown that prolonged sedentary time is related to negative health impacts in older populations, such as higher risks of metabolic syndrome, cardiovascular diseases, type 2 diabetes, reduced bone density, and all-cause mortality (5, 6). Given the negative health impacts

of sedentary behavior, it is worthwhile to further explore the factors associated with older adults' sedentary behavior in order to design effective behavioral change programs.

Manipulating neighborhood environments is a promising strategy for ensuring active aging and is anticipated to have forward-looking, long-lasting effects on the health-related behaviors of large amounts of older adults (7). For example, built environment characteristics (e.g., sidewalk availability and accessible public transportation) are related to physical activity (8) and active transport (9), pedestrian accidents (10), and several health-related behaviors (11, 12). In particular, older adults tend to spend more time in their own residential neighborhood than people in other age groups, and thus their health behaviors are more likely influenced by the neighborhood built environment (13). This may highlight the importance of developing effective strategies to reduce older adults' sedentary behavior through urban design and planning initiatives. In addition, most of the previous evidence was obtained using subjective environmental questionnaires, which capture different constructs of the street environmental characteristics than those measured by objective evaluations of environments (14). For example, street intersection density and sidewalk availability cannot be accurately determined through subjective measures. As such, a better understanding of objectively measured neighborhood environmental factors associated with sedentary behavior in older adults can be informative and of value in designing effective behavioral change programs. In addition, existing studies on this issue have investigated the relationship of neighborhood environmental attributes with total sedentary time (15, 16) or domain-specific sedentary behavior (17–19) in older adults. These studies have revealed important results for older adults (aged 65 years or above) regarding the relationship between neighborhood environment and sedentary behavior. However, these previous studies have been limited in that they have employed self-reported sedentary measures (17, 18) or total objectively measured sedentary time (15, 16). To enhance the evidence base used for advising policy and urban design initiatives, this study aims to prove the relationships between neighborhood environment and the patterns of objectively assessed sedentary behavior among older persons.

## Materials and Methods

### Participants

**Figure 1:**
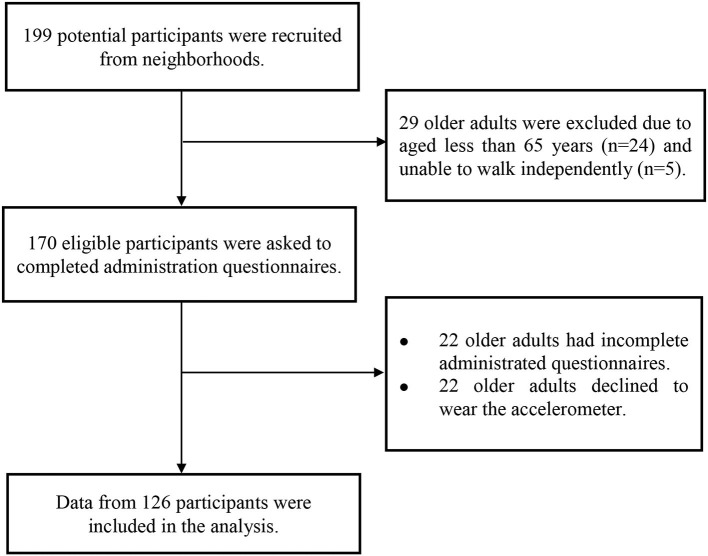
Flow chart of participant selection.

A total of 199 older adults (aged ≥65 years) who lived in the community were recruited from April to September 2018 in Taipei, Taiwan. The participants were recruited through local advertisements and announcements. Potential participants were ineligible if they were unable to walk (*n* = 5) or under 65 years of age (*n* = 24). In all, 170 participants completed the sociodemographic questionnaire with the assistance of a team of trained research assistants. Furthermore, each of the participants was asked to wear an accelerometer device for seven consecutive days. Of these, 22 participants declined to wear the accelerometer, and 22 participants had incomplete and/or missing data for the self-administered questionnaire. Finally, a total of 126 participants completed the questionnaire and also wore an accelerometer for seven consecutive days. Each participant who completed the questionnaire and wore the accelerometer for the full period of time requested received a convenience store voucher worth US$7. A flow diagram of the study recruiting process is presented as Figure 1. Signed informed consent from each of the participants was required before the participant took part in the study. We obtained ethical approval for the study from the Research Ethics Committee of National Taiwan Normal University (REC number: 201711HM003).

### Objectively Measured Sedentary Behavior

Triaxial ActiGraph (wGT3X-BT, Pensacola, FL, USA) accelerometer model was used to assess sedentary behavior. Participants were advised to carry the accelerometer on their waist, which recorded movement on three axes for ensuing 7 days. Valid data were collected from accelerometers worn by participants for at least 3 days with 1 weekend day. For each valid day, ≥600 min (≥10 h) of wear time is required, excluding sleep time. Following the method used in previous studies (20, 21), total sedentary time (time spent sitting, min/day), number of sedentary bouts ≥ 30 min (times/day), and duration of sedentary bouts ≥ 30 min (min/day) were calculated for the analysis. Each minute with an accelerometer count below 100 counts/min was considered sedentary time. The drop time of a sedentary bout was set at 2 min for data analysis. Accelerometer data were analyzed using ActiLife (software, version 6.13.3, Pensacola, FL, USA).

### Neighborhood Environmental Attributes

Geographic information system software (ArcGIS; ESRI, Redlands, CA) was used to assess neighborhood built environmental attributes, which refer to human-made surroundings, including houses, sidewalks, streets, leisure/utilization destinations, and public transportations. According to previous studies (22, 23), we included five neighborhood built environmental factors: (1) Resident density (number of population per square kilometer); (2) street intersection density (the number of intersections per square kilometer); (3) sidewalk availability (the sum of the areas (square meter) of a paved path of a road, according to the open data of the National Development Council of Taiwan (24); (4) accessible destination (the amount of 30 different types of destination, including convenience stores, supermarkets, hardware shops, fruit stores, dry cleaning stores, coin laundromats, clothing stores, post offices, libraries, book stores, fast food stores, cafés, banks, restaurants, video shops, video rental shops, pharmacies, drug stores, hairdressers, parks, gyms, fitness clubs, sports facilities, kindergartens, elementary schools, junior high schools, high schools, 2-year colleges, 4-year colleges, and universities in the residential village (25, 26); and (5) accessible public transportation [the amount of mass rapid transit (MRT) exits, train stations, high speed rail stations, and bus stops in the residential village]. We used each participant's geocoded residential neighborhood as a unit for calculating these five environmental measures of built environment, which has been reported as a valid scale (27). Please find the summary of neighborhood environmental attributes in **Table 2**.

### Statistical Analyses

Data were analyzed from 126 community-residence older adults who provided valid information in regard to the study variables. Chi-square tests were conducted to compare the differences of characteristics between included and excluded participants. Standard multiple linear regression, the enter method, was used to analyze the associations between neighborhood walkability attributes and the patterns of objectively measured sedentary behavior with adjustment for covariates [age, marital status, educational attainment, working status, living status, perceived health, body mass index (BMI), and accelerometer wear time]. All statistical data were analyzed with IBM SPSS, version 23.0 (SPSS Inc., IBM, Chicago, IL, USA). The significance level was set at *p* < 0.05.

## Results

Table 1 shows the characteristics of the sample. Chi-square tests showed proportional differences in age and marital status between included and excluded participants (data not shown). The mean age was 69.9 ± 5.0 years. A total of 126 participants (men, 36; women, 90) were included in this study. Most of the study population was married (65.9%) and lived with others (88.9%), had no University or higher education (78.6%), was not employed (96.8%), perceived health as poor (69.8%), and presented a BMI mean (SD) of 24.2 (3.4).

**Table 1:** Personal and accelerometer-related attributes of participants.

**Variables**	**Category**	**Total sample (*n* = 126), *N* (%)**
Age, *M* (SD)		69.9 (5.0)
BMI (kg/m^2^)		24.2 (3.4)
Gender	Men	36 (28.6)
	Women	90 (71.4)
Marital status	Married	83 (65.9%)
	Unmarried	43 (34.1%)
Living status	Living with others	112 (88.9%)
	Living alone	14 (11.1%)
Educational level	Tertiary education	27 (21.4%)
	No tertiary education	99 (78.6%)
Employment	Yes	4 (3.2%)
	No	122 (96.8%)
Perceived health	Good	38 (30.2%)
	Poor	88 (69.8%)
Wear time (min/day), *M* (SD)		920.5 (85.0)
Total sedentary time (min/day), *M* (SD)		603.8 (75.6)
Number of sedentary bouts (times/day), *M* (SD)		6.1 (2.0)
Sedentary bouts duration (min/day), *M* (SD)		273.3 (103.3)

Tertiary education: university or college degree or higher.

*N, number; M, mean; SD, standard deviation; BMI, body mass index*.

Table 2 shows the mean and standard deviation *M* (SD) of each neighborhood attributes. The *M* (SD) of resident density was 30594.27 (14698.35); street intersection density was 211.21 (92.66); sidewalk availability was 3603.10 (2704.94); accessible destination was 14.8 (11.73); and accessible public transportation was 23.00 (18.00).

**Table 2:** Summary of neighborhood environmental attributes.

**Attributes**	**Description**	***M* (SD)**
Resident density	The number of population per square kilometer	30594.27 (14698.35)
Street intersection density	The number of intersections per square kilometer	211.21 (92.66)
Sidewalk availability	The sum of the areas (square meter) of a paved path of a road	3603.10 (2704.94)
Accessible destination	The total amount of 30 destination types	14.8 (11.73)
Accessible public transportation	The amount of MRT exits, train stations, high speed rail stations, and bus stops	23.00 (18.00)

*M, mean; MRT, mass rapid transit; SD, standard deviation*.

Table 3 shows the results of the regression analysis of the categorical environmental attributes. After adjustment for potential confounders, only one objectively measured environmental attribute (sidewalk availability) was negatively associated with the number of sedentary bouts (β = −0.185; 95% CI: −0.362, 0.015; *p* = 0.034) and sedentary bout duration (β = −0.180; 95% CI: −0.354, −0.011; *p* = 0.037).

**Table 3:** Relationships between neighborhood environmental attributes and objectively measured sedentary behavior patterns in older adults.

**Objectively assessed attributes**	**Total sample**		
	**β**	**95% CI**	***p***
**Total sedentary time**			
Resident density	−0.005	(−0.167, 0.157)	0.949
Street intersection density	−0.032	(−0.193, 0.128)	0.689
Sidewalk availability	−0.140	(−0.298, 0.016)	0.078
Accessible destination	−0.089	(−0.246, 0.066)	0.257
Accessible public transportation	−0.102	(−0.260, 0.054)	0.196
**Number of sedentary bouts**			
Resident density	0.028	(−0.151, 0.208)	0.753
Street intersection density	−0.008	(−0.186, 0.169)	0.925
Sidewalk availability	**−0.185**	**(−0.362, 0.015)**	0.034^*^
Accessible destination	−0.124	(−0.297, 0.047)	0.154
Accessible public transportation	−0.136	(−0.310, 0.036)	0.119
**Sedentary bouts duration**			
Resident density	0.052	(−0.124, 0.229)	0.559
Street intersection density	0.023	(−0.152, 0.199)	0.790
Sidewalk availability	**−0.180**	**(−0.354**, **−0.011)**	0.037^*^
Accessible destination	−0.134	(−0.305, 0.034)	0.116
Accessible public transportation	−0.132	(−0.304, 0.037)	0.123

Adjusted for gender, age, marital status, educational level, working status, living status, perceived health, body mass index (BMI), and device wear time.

CI, confidence interval; SD, standard deviation.

* *p < 0.05*.

## Discussion

This study is the first to examine the relationships between objectively assessed neighborhood environmental attributes and the patterns of objectively assessed sedentary behaviors among urban community-residing older adults in Taiwan. We found that the availability of favorable neighborhood sidewalks was negatively related to both the number and duration of 30-min sedentary bouts in our sample. Our findings extend previous findings concerning this issue (15–18) and highlight the important role of neighborhood environments in older adults' sedentary behavior patterns. In terms of informing policies regarding healthy and age-friendly cities in Taiwan, our results could be taken to suggest that increasing sidewalk availability in neighborhoods could be an effective strategy for preventing older adults' prolonged sedentary behavior.

The mechanism underlying the relationship between sidewalk availability and older adults' prolonged sedentary bouts remains unclear since only a few studies have examined this issue. It is possible that neighborhood built environments with favorable sidewalk availability can assist older adults' mobility among their nearby surroundings (28), such as when taking walks from home for errands or to leisure destinations. Furthermore, it is also possible that neighborhoods with favorable sidewalk availability can enhance pedestrian safety in urban environments (29). Consequently, older adults might spend more time participating in physical activity in their neighborhoods, thereby avoiding prolonged bouts of sedentary behavior and having fewer bouts of sedentary behavior in general. Prospective studies are needed in the future, however, to further investigate the relationship between sidewalk availability and older adults' prolonged bouts of sedentary behavior.

There were several limitations to this study. First, our study was based on a cross-sectional design; accordingly, we were not able to draw a causal relationship between neighborhood environments and older adults' sedentary behavior. Second, the neighborhood environmental attributes for each participant were obtained using the location of each participant's residential neighborhood but not the participant's exact residential address. Most Taiwanese older adults are reluctant to disclose their exact residential address, which was the reason for this limitation (27). Nevertheless, the residential neighborhood has been widely used as a validated geographic unit for measuring walkability attributes in neighborhoods (30). Third, other environmental attributes that may be related to physical activity and sedentary behavior, such as green spaces and public open spaces, were not examined in the present study. Future studies examining such attributes are warranted. Finally, the sample of our study was limited by the restricted number of participants, who mostly consisted of female participants living in urban settings.

## Conclusion

This study is the first to find that in urban areas, favorable neighborhood environments are negatively associated with sedentary behavior patterns in a sample of community-dwelling older Taiwanese adults. As such, neighborhood environments with favorable sidewalk availability could be supportive in preventing older adults' prolonged bouts of sedentary behavior. These findings are critical for informing environmental policy initiatives to prevent sedentary lifestyles among older adults.

## Data Availability Statement

The raw data supporting the conclusions of this article will be made available by the authors, without undue reservation.

## Ethics Statement

The studies involving human participants were reviewed and approved by the Research Ethics Committee of the National Taiwan Normal University. The patients/participants provided their written informed consent to participate in this study.

## Author Contributions

Conceptualization was carried out by S-HC, M-CH, SW, and YL. The methodology was provided by RR, M-CH, and Y-CY. The software was obtained by RR, M-CH, and Y-CY. The investigation was conducted by S-HC and MH. Resources were provided by S-HC, M-CH, J-HP, and YL. Data curation was performed by RR, M-CH, and SW. Writing–original draft preparation was done by S-HC, RR, Y-CY, and YL. Writing–review and editing were done by S-HC, RR, M-CH, SW, J-HP, and YL. Supervision was performed by S-HC, M-CH, and J-HP. Funding acquisition was performed by S-HC, M-CH, J-HP, SW, and YL. All authors contributed to the article and approved the submitted version.

## Conflict of Interest

The authors declare that the research was conducted in the absence of any commercial or financial relationships that could be construed as a potential conflict of interest.
